# The Female Brain Health and Endocrine Research in Africa Study (FemBER‐AFRICA): Identifying endocrinological, lifestyle, psychosocial and socio‐cultural targets for Alzheimer’s disease prevention in women of African ancestry

**DOI:** 10.1002/alz.093358

**Published:** 2025-01-09

**Authors:** Chinedu Udeh‐Momoh, Rachel W Maina, Edna N Bosire, Linda N Khakali, Sarah Gregory, Karen Blackmon, Thomas Thesen, Dimitra Kafetsouli, Samuel Gitau, Anne Nyambura Njogu, Cynthia Smith, Sylvia Mbugua, Sheila Waa, Jasmit N Shah, Kendi Muchungi, Tanisha G Hill‐Jarrett, Elena Tsoy, Zul Merali, Jennifer S. Yokoyama, Alina Solomon, Graciela Muniz‐Terrera, Michelle M. Mielke, Tamlyn J Watermeyer

**Affiliations:** ^1^ Wake Forest University School of Medicine, Winston‐Salem, NC USA; ^2^ Aga Khan University, Nairobi Kenya; ^3^ Female Brain Health and Endocrine Research (FemBER) Consortium, Newcastle, Edinburgh, London United Kingdom; ^4^ Scottish Brain Sciences, Edinburgh, Lothian United Kingdom; ^5^ Imperial College, London United Kingdom; ^6^ University of California, San Francisco, San Francisco, CA USA; ^7^ Department of Clinical Medicine/Neurology, University of Eastern Finland, Kuopio, Finland, Kuopio Finland; ^8^ Department of Social Medicine, Ohio University, Athens, OH USA; ^9^ University of Edinburgh, Edinburgh United Kingdom; ^10^ Faculty of Health & Life Sciences, Northumbria University, Newcastle United Kingdom

## Abstract

**Background:**

The Global Dementia Action Plan 2017‐2025 specifies key targets, with an emphasis on building research infrastructure and capability across the Global South. However, to date, only 0.1% of total research in Africa constitutes dementia research, the lowest volume of all LMIC regions. Several biomarker and risk models of the preclinical and prodromal stages of Alzheimer’s disease (AD) are proposed as optimal junctures for disease prevention, yet they ignore the influence of biological sex/gender on cognitive and disease processes, despite female sex/gender constituting a major AD modifier. Similarly, the application of these models in ethno‐racial and culturally diverse cohorts is limited, further compromising their generalizability. Consequently, there exists little understanding of the intersections of sex/gender, ethno‐racial, culture, and other risk factors in AD and related dementias (ADRD) onset.

**Method:**

This partnership is led by The Brain and Mind Institute, Aga Khan University Nairobi, Kenya. We aim to i) establish a highly‐phenotyped co‐designed readiness cohort in a socio‐economically diverse community in Kenya (n = 250, ≥35 y/o) of individuals across the AD clinical continuum; with a focus on characterizing sex differences in psychobiological determinants of AD and ii) compare harmonized data from our cohort with western cohorts enriched with diasporic African populations to determine culturally‐specific, ethno‐racial, lifestyle, health and biological determinants between indigenous and diasporic Africans and other ethnicities.

**Result:**

We extend Nigerian‐based Ibadan study pilot data, that identified female sex as a significant factor associated with higher AD risk (HR:1.51, 95% CI: 1.67‐ 1.36), with sex‐specific differences in AD risk profiles noted. Population characteristics will be defined for the initial recruitment phase for FemBER‐Africa (target n = 50 by July 2024), purposively sampled from the Nairobi locality to ensure balanced groups by sex/gender, ethno‐racial characteristics, age, socio‐economic status. Learnings from the cultural adaption of tools and measures will also be presented.

**Conclusion:**

FemBER‐Africa addresses knowledge gaps surrounding the intersectionality of sex/gender, ethno‐racial, culture and psychobiological determinants of brain health in indigenous and diasporic African populations. This readiness cohort represents a world‐first culturally informed African ADRD program and an optimal platform for future culturally specific risk‐reduction and prevention trials, adaptable for other African contexts.

